# Monitoring Pertussis Infections Using Internet Search Queries

**DOI:** 10.1038/s41598-017-11195-z

**Published:** 2017-09-05

**Authors:** Yuzhou Zhang, Gabriel Milinovich, Zhiwei Xu, Hilary Bambrick, Kerrie Mengersen, Shilu Tong, Wenbiao Hu

**Affiliations:** 1 School of Public Health and Social Work; Institute of Health and Biomedical Innovation, Queensland University of Technology, Brisbane, Queensland Australia; 2 Science and Engineering Faculty, Mathematical and Statistical Science, Queensland University of Technology, Brisbane, Queensland Australia; 3 School of Public Health and Institute of Environment and Human Health, Anhui Medical University, Hefei, China; 4 Shanghai Children’s Medical Centre, Shanghai Jiao-Tong University, Shanghai, China

## Abstract

This study aims to assess the utility of internet search query analysis in pertussis surveillance. This study uses an empirical time series model based on internet search metrics to detect the pertussis incidence in Australia. Our research demonstrates a clear seasonal pattern of both pertussis infections and Google Trends (GT) with specific search terms in time series seasonal decomposition analysis. The cross-correlation function showed significant correlations between GT and pertussis incidences in Australia and each state at the lag of 0 and 1 months, with the variation of correlations between 0.17 and 0.76 (p < 0.05). A multivariate seasonal autoregressive integrated moving average (SARIMA) model was developed to track pertussis epidemics pattern using GT data. Reflected values for this model were generally consistent with the observed values. The inclusion of GT metrics improved detective performance of the model (β = 0.058, p < 0.001). The validation analysis indicated that the overall agreement was 81% (sensitivity: 77% and specificity: 83%). This study demonstrates the feasibility of using internet search metrics for the detection of pertussis epidemics in real-time, which can be considered as a pre-requisite for constructing early warning systems for pertussis surveillance using internet search metrics.

## Introduction

Pertussis is one of the ten mosy common infectious diseases, having the highest risk of death worldwide^1^. While pertussis is a vaccine preventable disease, it remains endemic and is responsible for around 45 million new cases and approximately 297,000–409,000 deaths annually^2^. In Australia, pertussis notification by clinic doctors, health organizations and child-care facilities has been mandatory since 1991. Incidence rates of pertussis have increased from 1.8 cases per 100,000 people in 1991, peaking in 2009 at 127.8 cases per 100,000 people^3^. Conventional infectious diseases surveillance systems rely on case reporting or disease condition submissions to the relevant public health authority^4^, which can take up to 2 weeks from the onset of events to the detection of events^5^. This lag in reporting limits the ability of conventional surveillance systems to provide intelligence and implement action, particularly in the early phases of outbreaks^6^.

The increase in trends of pertussis infections since the implementation of mandatory notification has demonstrated the need to develop better systems for identifying emerging outbreaks; this requires the development of new approaches^7^. Internet-search metrics has been seen as a basis for surveillance and early warning systems for epidemics^8, 9^. This new tool relies on the premise that people who contract a disease will actively seek information about their condition from the internet and that disease activity can be estimated by tracking changes in frequencies of related internet searches for key terms. Internet search metrics are able to reflect disease activity of larger fraction of the community and generate timely disease information through targeting people in the early phase of disease process^10^. For instance, a successful prediction for Zika outbreak (1–3 weeks ahead) than that which is predicted by traditional surveillance systems has been achieved in 2016 through analysing Zika-related web queries in Latin America^11^. Moreover, the potential for internet-based surveillance systems to be incorporated into and bolster the capacity of conventional surveillance systems was shown by Scarpino and colleagues^12^. The resulting networks which incorporate Google Flu Trends (GFT) not only better predicted influenza associated hospitalizations and included less providers than the existing influenza surveillance system, but were also shown to enhance traditional, provider-based surveillance systems^12^.

Australia is in an ideal location to assess the ability of internet based surveillance systems. Because internet penetration in Australia is very high by world standards; 84.6% of Australians have access to the internet^13^, and 74% of internet users in Australia reported that they have used internet to search health or medical-related information^14^. Furthermore, internet use in Australia is currently dominated by a single search engine. Google dominates Australian internet search engine market with a market share of more than 90% in 2013^15^. These features make collection of representative data easier in Australia than for many other locations.

The correlation between internet search metrics and pertussis notifications in Australia was studied previously in 2014^10^. The results of the study indicated that internet search metrics have a potential role in forecasting emerging infectious disease events, especially for vaccine-preventable diseases, such as pertussis. However, the value of internet search metrics in reflecting the incidence rate of pertussis in Australia has not yet been fully examined. This study aims to assess whether internet search queries are a useful data source for monitoring pertussis epidemics in real-time and explores the development of an empirical model to detect the pattern of pertussis using internet search metrics in Australia.

## Results

### Descriptive Analysis of Pertussis Incidence rates and Google Trends (GT) data

**Figure 1:**
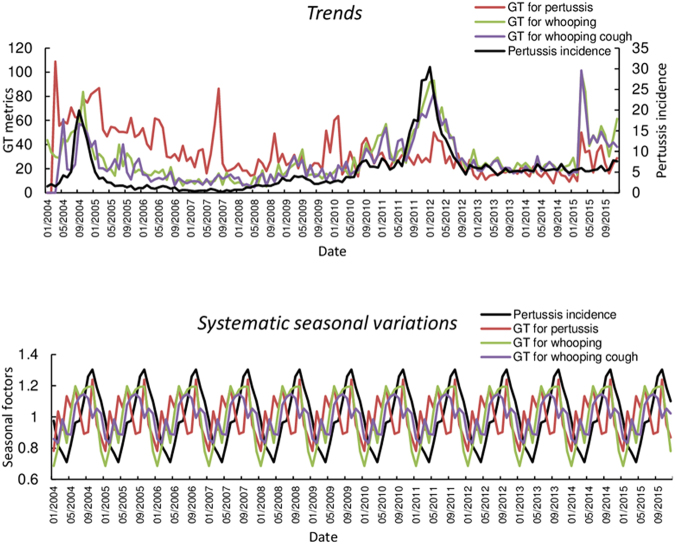
Systematic seasonal variations and trends for pertussis incidence rates and GT at national level from 2004 to 2015.

The terms “pertussis”, “whooping” and “whooping cough” were chosen for analysis, as “whooping” and “whooping cough” are the two most common symptoms in pertussis cases^16^. Analyses of systematic seasonal variations and trends for the Australian National Notifiable Diseases Surveillance System (NNDSS) pertussis incidence rates and GT metrics in Australia and each state are presented in Figs 1 and S1. Clear seasonal patterns were evident in both national and state level data over the study period. The data were observed to peak between June and November, and to trough from October to April. Trends in pertussis incidence and search metrics data were further analysed by removing systematic seasonal variations. There are increasing trends in pertussis incidence and GT metrics at the national and state level with fluctuations over the study period. However, the decreased trends were observed in the State of Tasmania (Tas) where pertussis incidence and GT metrics were observed to peak between 2009 and 2012 at both national and state level.

The results of Spearman’s correlations indicated that pertussis incidence rates were positively correlated with GT for pertussis in Australia during the overall study period. The strongest correlation was observed between GT for whooping cough and pertussis incidence rates with a correlation coefficient of 0.82 (p < 0.01) (Table S1).

### Time-series cross correlation analysis

**Figure 2:**
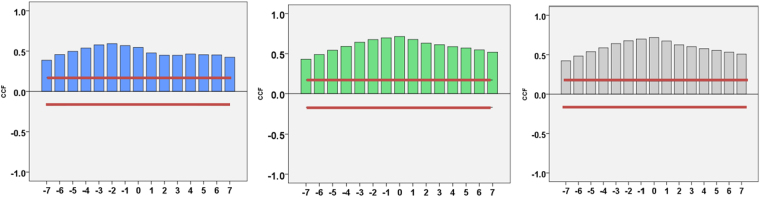
Coloured bars show time-series cross correlation results for pertussis incidence rates with GT metrics (2004–15) in Australia. Blue bars indicate the value of search term pertussis, the values of search queries whooping and whooping cough are indicated by green bars and grey bars separately. Confidence intervals (95%) are indicated by the solid red lines (X axis: lag value, Y axis: CCF value).

Time-series cross correlation analysis demonstrated that NNDSS monthly pertussis incidence rates to be positively correlated with monthly GT metrics for selected search terms in Australia (Fig. 2). However, the variation of positive correlations between pertussis incidence rates and GT for search terms were observed by states and territories (Fig. S2). Correlations between pertussis surveillance and GT at national and state levels ranged from 0.17 to 0.76 (p < 0.05) (Table S2). Much larger positive correlations were generally observed for pertussis incidence rates correlating at lags of 0 or 1 month (lag value 0 or −1) with GT in Australia and each state (Table S2). Lag values should be interpreted as product–moment associations between two time series; they reckon the first time series can be related to the second time series by identifying a series of temporal offsets between two time series. For example, a lag value of −1 indicates that the first series (NNDSS pertussis surveillance data) is shifted backwards one unit (a month) when cross correlations were assessed performing time series data. In contrast, the primary series is shifted forward one unit if a lag value of 1 is used. Moreover, the GT for “whooping” and “whooping” cough were generally correlated with pertussis incidence rates more strongly than that for the term “pertussis”.

### Seasonal autoregressive integrated moving average (SARIMA) model

We used a SARIMA model with GT to develop a forecast model. The GT metrics for the search term whooping cough was selected as the independent variable, since they had the highest Spearman’s rho values with pertussis incidence rates in Australia (r = 0.82) (Table S1). The SARIMA model (2,0,2) (1,0,0) correlated to the GT metrics was found to offer the best fit to the data. The goodness-of –fit of the model was checked by calculating the autocorrelation function (ACF) and partial autocorrelation (PACF) in the residuals. The results of residual series of the SARIMA model (2,0,2) (1,0,0) are presented in Figure S3 (Fig. S3). The results of the ACF and PACF demonstrated autocorrelations and partial autocorrelations of this model to fluctuate randomly near zero. Therefore, the analysis of goodness-of-fit revealed that the SARIMA model (2,0,2) (1,0,0) fitted the data well. The R^2^ for the SARIMA model that excluded GT metrics was 93% and the BIC values was 0.93. The model that incorporated GT metrics performed better with the larger R² and smaller BIC values (95% and 0.70 respectively). Thus, the model that incorporated GT data was selected as the preferred predictive model for the period of January 2013 to December 2015. Results of SARIMA models are presented in Table 1.

**Table 1:** Parameters estimates and their testing results of the SARIMA (2,0,2) (1,0,0) model.

Parameters	Coefficients	Standard error	t	P value
AR	0.91	0.23	4.01	0.000
SAR	0.31	0.11	2.95	0.004
GT	0.06	0.01	5.66	0.000

SARIMA: Seasonal Autoregressive Integrated Moving Average Model, AR: autoregressive, GT: Google Trends metrics for search term whooping cough.

**Figure 3:**
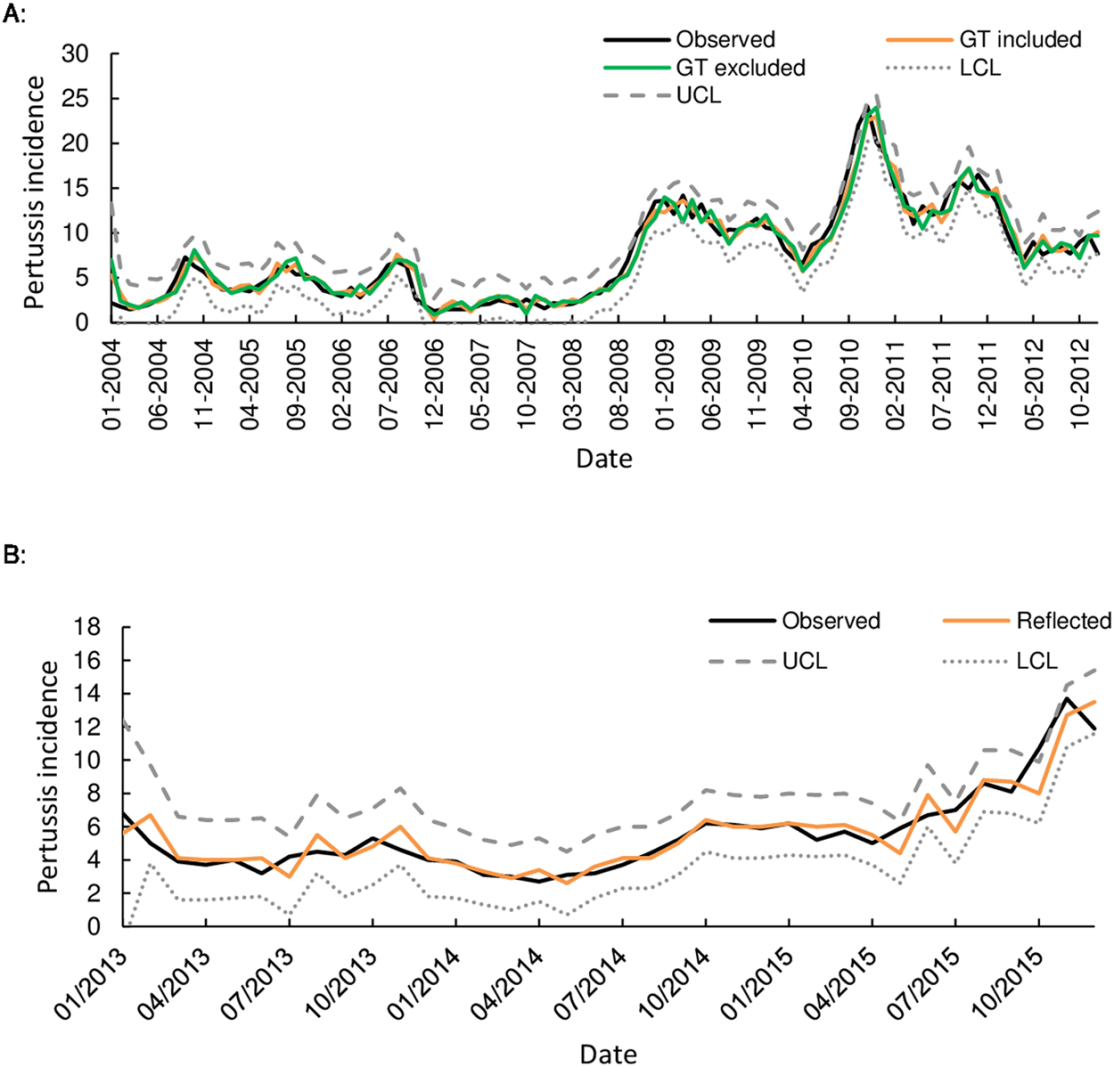
(**A**) Observed and fitted value of the SARIMA models from 2004 to 2012. UCL and LCL are presented for the GT included model. (**B**) Reflected pertussis incidence rates between 2013 and 2015 based on the SARIMA model.

The selected model was used to reflect the monthly pertussis notification rates at the Australian national level for the 36 months from 1^st^ January 2013 to 31^st^ December 2015, and was validated by the NNDSS pertussis notification rates data (Fig. 3). The validation for the 36 months showed a good fit between the NNDSS pertussis notification rates and reflected pertussis notification rates using the SARIMA model (2,0,2) (1,0,0) with analysis indicating an overall agreement of 81% (sensitivity: 77% and specificity: 83%) (Table S3).

## Discussion

Seasonal decomposition analysis of pertussis notification rates and GT showed clear seasonal patterns at both national and state and territory levels. Larger pertussis incidence rates and GT index number were observed during winter and spring months in the study period. Furthermore, there are similar seasonal patterns and trends between pertussis incidence rates and GT metrics for “pertussis”, “whooping” and “whooping cough”. The findings of this study support the hypothesis that GT is a valuable data source for detecting pertussis infections in real-time.

Pertussis is recognised as a disease of increasing concern in Australia; incidence was observed to increase in all states except in Tasmania. Although Australia has run a pertussis vaccination programme for decades, with high national coverage^17^. The increased incidence may partly result from the currently used acellular vaccine which is transited from whole-cell pertussis vaccines. A study reported that the level of protection of acellular vaccine is lower than that of the previously used whole-cell vaccine^18^. Additionally, the shift to PCR testing, which is more sensitive than early diagnostic tests and evolution of *B. pertussis* may also contribute to the increase in the outbreaks of pertussis in Australia^17, 19^. Other factors that contribute to this epidemic will require further studies, especially for Tasmania. However, analyses were unable to be performed for Northern Territory (NT). The reason may be because the NT has the least population size and pertussis incidence of any state in Australia. Thus, the GT and pertussis data size was inadequate to perform data analysis.

In this study, three search queries were analysed and trends were correlated with the NNDSS data for pertussis incidence; the significance of the associations was variable by states. Income and education levels have previously been identified as factors affecting internet use^20^. The state that had higher percentage of youth (15–19 Years) engagement in studying full-time generally correlated with the pertussis incidence more strongly^21^. Additionally, the GT for search queries “whooping” and “whooping cough” had stronger associations than “pertussis” when correlated with NNDSS pertussis incidence. “Whooping” and “whooping cough” are the two most common symptoms in pertussis cases. People recognise that pertussis has a distinctive cough and is of particular concern to a defined population (young children). As such, they are likely to notice it in this scenario. Additionally, the majority of the population has never heard of “pertussis” as it is an academic word, whereas most people know what “whooping” and “whooping cough”, since these terms are colloquial. Therefore, these two search queries are more popular among pertussis patients. Furthermore, changing health-seeking behaviour and regional culture can also affect the popular search terms^22^.

The purpose of using internet-based data for monitoring an interest area is not usually to predict the occurrence of pertussis event in the future, but aims to collect information in a more timely fashion. This application can be seen as a “nowcasting” of diseases events^23^. In the study, significant positive correlations are exhibited at both national and state level at 0–2 lag months. This result can be seen as an indicator of how much faster internet metrics may collect the data, which can be considered as a pre-requisite for constructing early warning systems for pertussis using GT. The signalling of variation in GT metrics may provide sufficient time for government and health authorities to implement pertussis preventive measures such as pertussis-related education and disease control, and therefore may be directly used for policy-making and decision-making process in the monitoring and control of pertussis infection.

The SARIMA model has been widely used to reflect the incidence rates of various infectious disease, such as dengue fever, malaria and hepatitis E^24–26^. In this study, a SARIMA model was developed at the national level using NNDSS pertussis surveillance and GT data between January 2004 and December 2012 and then used to detect pertussis incidence rates from January 2013 to December 2015. The result of this study suggests that the SARIMA model (2,0,2) (1,0,0) to be the most appropriate model. Furthermore, the SARIMA model that included internet search metrics provided a better fit to NNDSS pertussis incidence rates than the model that excluded these metrics. This model has the potential for reflecting pertussis case number using GT data for any given number of future time intervals. Thus, the incorporation of internet search metrics based models into traditional surveillance systems has the potential to bolster capacity in the monitoring of pertussis. Internet search metrics have the potential to be used not just to enumerate the magnitude of outbreaks by monitoring the trends of GT, but also to identify high risk areas providing health authorities with strategic instruments for health resource management and allocation in those areas which have higher GT metrics.

There are two limitations in the study presented here. First, the accuracy of GT may be influenced by different levels of access to the internet^7^. Amongst states and territories in Australia in 2013, household proportions of internet access at home ranged from 89% in the Australian Capital Territory to 78% in Tasmania, and the proportion of Australian households with internet access was higher for those located in capital cities (85%), compared to those located outside of capital cities (79%)^27^. Second, it is acknowledged that there are different internet-seeking behaviours, self-reporting and media-driven bias between different sectors of community^7^. Previous studies reported that media bias can adversely impact internet-based surveillance systems^28^. For instance, GFT predicted more than double the peak of influenza-like illness (ILI) cases than the Centers for Disease Control and Prevention (CDC) in 2013^29^. A major reason for the overestimation may be the widespread media coverage of the severe flu season in 2013, which may have triggered many flu-related searches by people who were not ill^30^. However, a previous study used Autoregression model with Google search data to capture changes in people’s online search behaviour over time. These authors suggested that this approach could reduce the predictive errors^31^.

The potential of search query-based surveillance systems for monitoring infectious diseases has been reported in Australia for influenza. Boyle and colleagues demonstrated that GFT was strongly correlated with emergency department presentations or hospital admissions for influenza-like illness in Queensland between 2006 and 2009 (r = 0·35, 0.88, 0.91, 0.76 for 2006–2009 separately)^32^. The results of our study illustrate the potential for internet search metrics in the surveillance of pertussis in Australia. Such systems have the potential for facilitating government, health authorities and the public to respond to pertussis outbreaks. Future studies should explore ways to integrate internet-based approached into existing pertussis surveillance systems to expand capacity of existing ones in Australia. In the future, a dynamic and integrated spatiotemporal pertussis early warning system developed through web search engine query data combined with socio-environmental factors and historic disease surveillance data may have the potential to assist risk managers and local public health authorities to identify high risk communities.

## Methods

### Collection of Pertussis Surveillance Data

Pertussis surveillance data were collected from the NNDSS, which is available from the official website of Australian Government Department of Health (DoH). Both confirmed and probable pertussis cases are reported to the system by the state and territory health authority. The monthly notification rates of pertussis (per 100,000 population) were downloaded for the period of 1^st^ January 2004 to 31^st^ December 2015.

### Search Term Selection and Collecting Search Trend Data

The results of previous studies demonstrated that the internet search metrics for terms of “pertussis”, “whooping” and “whooping cough” were strongly correlated with pertussis surveillance data^10, 33^. Therefore, the terms of “pertussis”, “whooping” and “whooping cough” were chosen for analysis. The frequencies of search terms were collected via GT (www.google.com/trends/). GT computes the weighted sum of searching frequency for a search query based on its monthly search volume on the Google website, presenting the frequency as a normalised data series with values ranging from 0 to 100 (with 100 representing the point with the highest search frequency and other points scaled accordingly)^10^. For exporting the data of search term frequency, a.CSV file is provided by GT webpage, which indicates the search volume through the normalised data series with values ranging from 0 to 100. CSV files for search terms during January 2004 to December 2015 were downloaded from the GT website at national and state levels to collect monthly GT data.

### Data Analysis

#### Descriptive Analysis of Pertussis Incidence rates and GT data

Pertussis notification rates and GT data for selected internet search terms were combined in one spreadsheet using Microsoft Excel. All data analyses were performed through using IBM SPSS Statistics software, version 23. Statistical significance was set at P < 0.05. All data was checked for completeness and accuracy before analysis.

Decomposition procedures are able to describe the trend and seasonal factors in a time series. The goal of this analysis was to determine systematic seasonal variations in pertussis notification rates and GT metrics in the study period. In addition, the trend in pertussis incidence rates and GT metrics during the overall study period were identified by removing any systematic seasonal variations. Seasonal variations can be used to create and present seasonally adjusted values, which influence pertussis incidence rates and GT metrics trends in the study period. Thus, these trends can be seen more clearly when seasonal effects are removed from the trends.

#### Time-series cross correlation analysis

The Spearman’s rank correlation coefficient was used to assess the relationship between pertussis epidemic trends and GT at both national and state levels. The Spearman’s rank correlation is a nonparametric product-moment correlation, which can measure the strength of the association between pertussis occurrence trends and internet search trends. The correlation between these two variables was strong when a similar rank between the two variables was observed, in contrast, a weak correlation was observed when the two variables had a dissimilar rank.

To assess the correlations between NNDSS pertussis notification rates and GT metrics more specifically, time-series cross correlation was used in the study. This aimed to assess linear association between pertussis occurrence and GT search indices at national and state levels.

#### The SARIMA Model

Generally, there are three significant components of a SARIMA model, namely autoregressive (AR), differencing and moving average (MA). Three parameters are typically selected when fitting this model: (p, d, q); where p is the order of the AR, d is the order of the differencing, and q is the order of the MA^34^. The orders of the AR and MA models were identified by the analysis of autocorrelation function (ACF) and partial autocorrelation function (PACF). The selection of parameters of the model was based on the results of maximum likelihood. The goodness-of-fit of the model was checked by calculating autocorrelation for the residuals. In addition, the Bayesian Information Criteria (BIC) and the stationary R square (R²) were also used to examine the goodness-of-fit of the model. A SARIMA model can be considered as a good model if it has a large R² value and a small BIC value. A SARIMA model for pertussis incidence rates in Australia was developed by using the number of national monthly pertussis notification rates as the dependent variable and Australian GT for the search term which correlated to pertussis surveillance most strongly as the independent variable. To analyse the predictive capacity of the model, the data file was divided into two data sets: data from 1^st^ January 2004 to 31^st^ December 2012 data set (108 months in total) was used as a training set to construct a SARIMA model and data from 1^st^ January 2013 to 31^st^ December 2015 (36 months in total) was used as a test data set to validate the model. The model reflection (2013–2015) was performed using the data set between 1^st^ January 2004 and 31^st^ December 2012. The performance of SARIMA models with and without GT was also compared. The better model was select as the predictive model. The definition of outbreak in the study is when the pertussis incidence rate exceeded the median of the incidence rate in Australia over the study period.

## Electronic supplementary material

Supplementary information
