# Supplementary material for: Monitoring Pertussis Infections Using Internet Search Queries
Source: Sci Rep. 2017 Sep 5;7:10437. doi: 10.1038/s41598-017-11195-z (PMC5585203; doi:10.1038/s41598-017-11195-z)
Supplement: Supplementary file 1 — Supplementary information [file 41598_2017_11195_MOESM1_ESM.pdf]

# Supplementary Information

## Monitoring Pertussis Infections Using Internet Search Queries

Yuzhou Zhang[1], Gabriel Milinovich[1], Zhiwei Xu[1], Hilary Bambrick[1], Kerrie Mengersen[2], Shilu Tong[1,3,4], Wenbiao Hu[1]

1. School of Public Health and Social Work; Institute of Health and Biomedical Innovation, Queensland University of Technology, Brisbane, Queensland, Australia. Emails: Yuzhou Zhang: yuzhou.zhang@hdr.qut.edu.au; Gabriel Milinovich: g.milinovich@uq.edu.au; Zhiwei Xu: xzw1011@gmail.com; Hilary Bambrick: h.bambrick@qut.edu.au; Shilu Tong: s.tong@qut.edu.au; Wenbiao Hu: w2.hu@qut.edu.au

2. Science and Engineering Faculty, Mathematical and Statistical Science, Queensland University of Technology, Brisbane, Queensland, Australia. Kerrie Mengersen: k.mengersen@qut.edu.au

3. School of Public Health and Institute of Environment and Human Health, Anhui Medical University, Hefei, China. Shilu Tong: s.tong@qut.edu.au;

4. Shanghai Children's Medical Centre, Shanghai Jiao-Tong University, Shanghai, China. Shilu Tong: s.tong@qut.edu.au;

### Author contributions

W.H. developed the original idea for this research. Y.Z. collected, analysed, interpreted the data and drafted this manuscript. G.M., Z.X., K.M. contributed to revising the manuscript. H.B., S.T. reviewed the manuscript.

### Corresponding author

Wenbiao Hu, School of Public Health and Social Work; Institute of Health and Biomedical Innovation, Queensland University of Technology, Brisbane, Queensland, Australia. Phone: +61 31385724 Email: w2.hu@qut.edu.au;

## Appendix Content


| Section | | Page |
|---|---|---|
| Figure S1 | Systematic seasonal variations and trends for pertussis incidence rates and GT at state and territory level from 2004 to 2015; In Systematic seasonal variations graph: Y axis is Seasonal factors; In Trends graph: Y axis are GT metrics (left Y axis) and Pertussis incidence (right Y axis). | 24 |
| Figure S2 | Figure S2. Coloured bars show time-series cross correlation results for pertussis incidence rates with GT metrics (2004–15) in Australian states and territories. Blue bars indicate the value of search term pertussis, the values of search queries whooping and whooping cough are indicated by green bars and grey bars separately. Confidence intervals (95%) are indicated by the dashed lines (X axis: lag value, Y axis: CCF value). | 27 |
| Figure S3 | Autocorrelation function (ACF) and partial autocorrelation function (PACF) of the residual series of the SARIMA model (2,0,2) (1,0,0). | 29 |
| Table S1 | Spearman's rho values for the monthly GT metrics in Australia from 1 January, 2004 to 31 December, 2015. | 30 |
| Table S2 | Time-series cross correlation analysis in 1-2 lag months between the NNDSS pertussis surveillance data and GT from 2004 to 2015. | 31 |
| Table S3 | Sensitivity and specificity of the SARIMA model (2,0,2) (1,0,0) for pertussis. | 32 |

***Figure S1:*** Systematic seasonal variations and trends for pertussis incidence rates and GT at state and territory level from 2004 to 2015; In Systematic seasonal variations graph: Y axis is Seasonal factors; In Trends graph: Y axis are GT metrics (left) and Pertussis incidence (right).

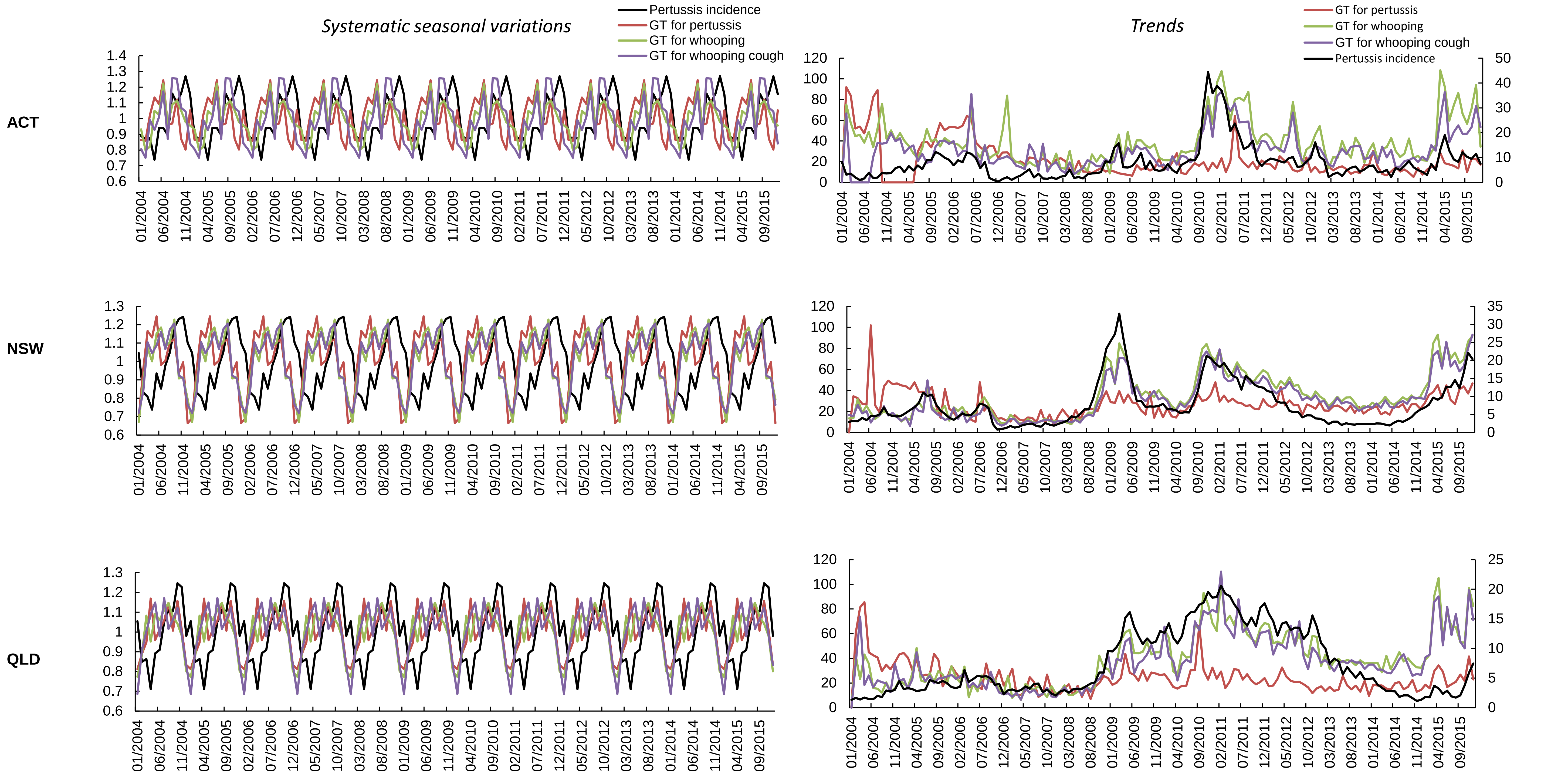

SA

TAS

VIC

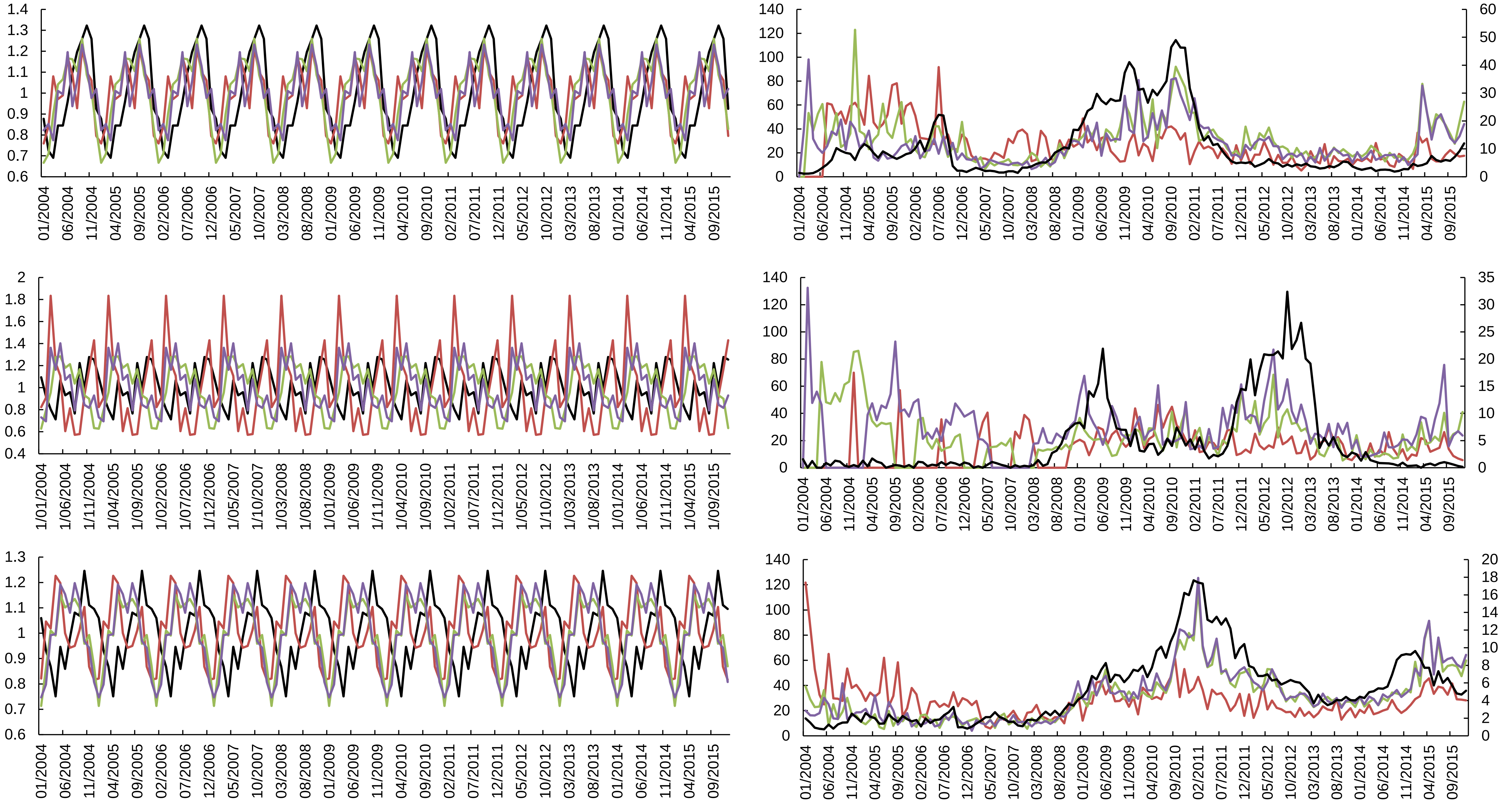

1.4
1.3
1.2
1.1
1
0.9
0.8
0.7
0.6
140
120
100
80
60
40
20
0
60
50
40
30
20
10
2
1.8
1.6
1.4
1.2
1
0.8
0.6
0.4
35
30
25
15
5
1.3
1.2
1.1
1
0.9
0.8
0.7
0.6
18
16
14
12
8
6
4
2
01/2004
06/2004
11/2004
04/2005
09/2005
02/2006
07/2006
12/2006
05/2007
10/2007
03/2008
08/2008
01/2009
06/2009
11/2009
04/2010
09/2010
02/2011
07/2011
12/2011
05/2012
10/2012
03/2013
08/2013
01/2014
06/2014
11/2014
04/2015
09/2015
1/01/2004
1/06/2004
1/11/2004
1/04/2005
1/09/2005
1/02/2006
1/07/2006
1/12/2006
1/05/2007
1/10/2007
1/03/2008
1/08/2008
1/01/2009
1/06/2009
1/11/2009
1/04/2010
1/09/2010
1/02/2011
1/07/2011
1/12/2011
1/05/2012
1/10/2012
1/03/2013
1/08/2013
1/01/2014
1/06/2014
1/11/2014
1/04/2015
1/09/2015

**WA**

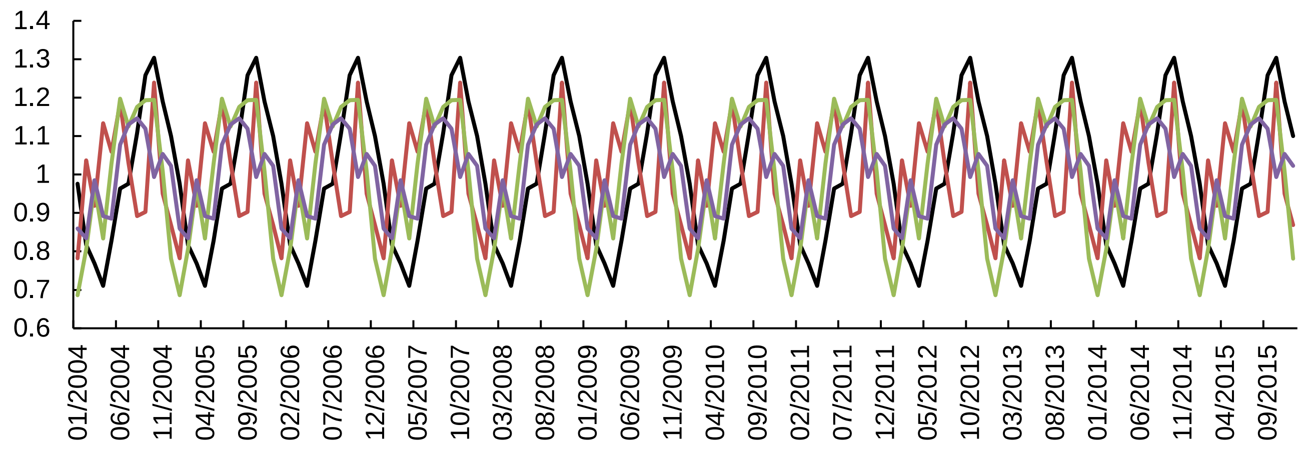


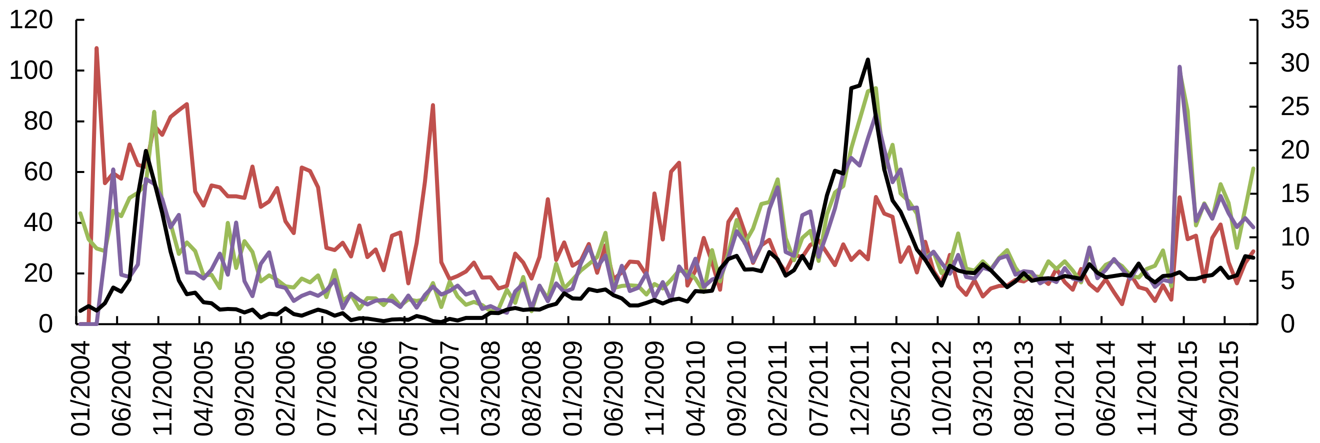


ACT : Australian Capital Territory; NSW: New South Wales; QLD: Queensland; SA: South Australia; TAS: Tasmania; VIC: Victoria; WA: Western Austral

**Figure S2.** Coloured bars show time-series cross correlation results for pertussis incidence rates with GT metrics (2004–15) in Australian states and territories. Blue bars indicate the value of search term pertussis, the values of search queries whooping and whooping cough are indicated by green bars and grey bars separately. Confidence intervals (95%) are indicated by the dashed lines (X axis: lag value, Y axis: CCF value).

- **ACT**

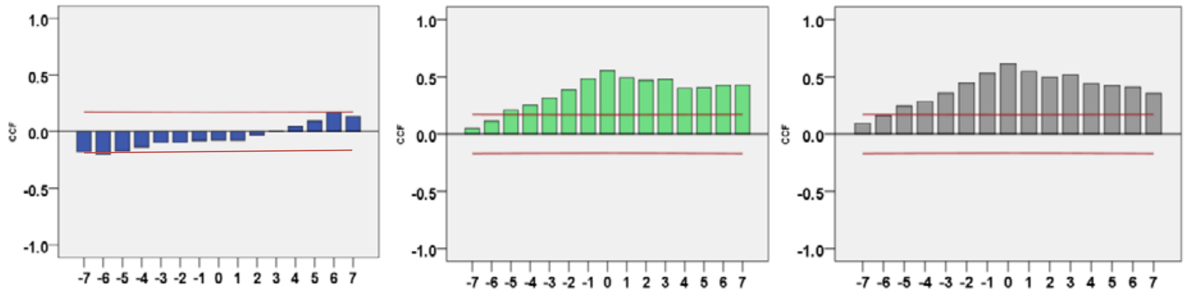


- **NSW**

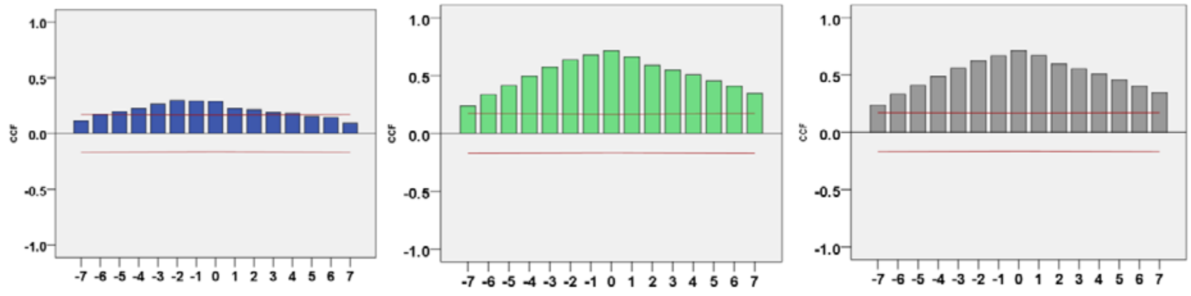


- **NT**

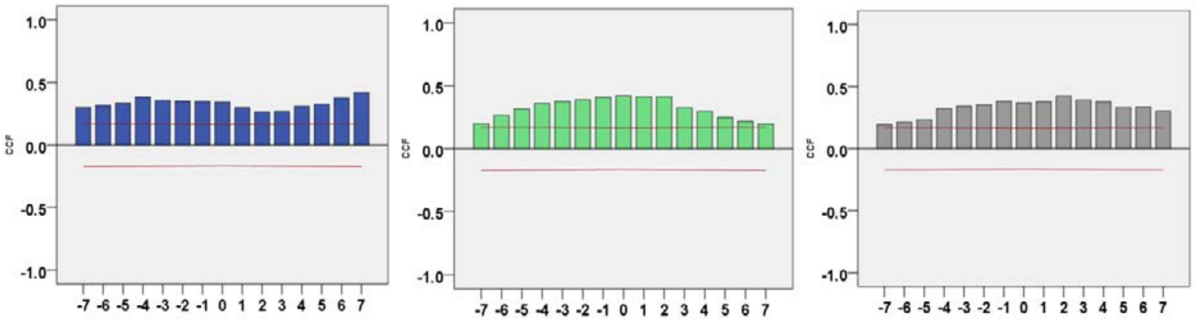


- **Qld**

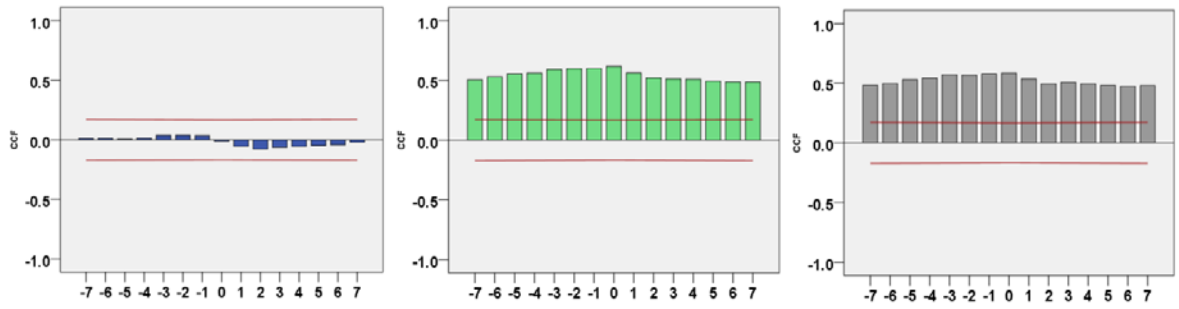

- **SA**

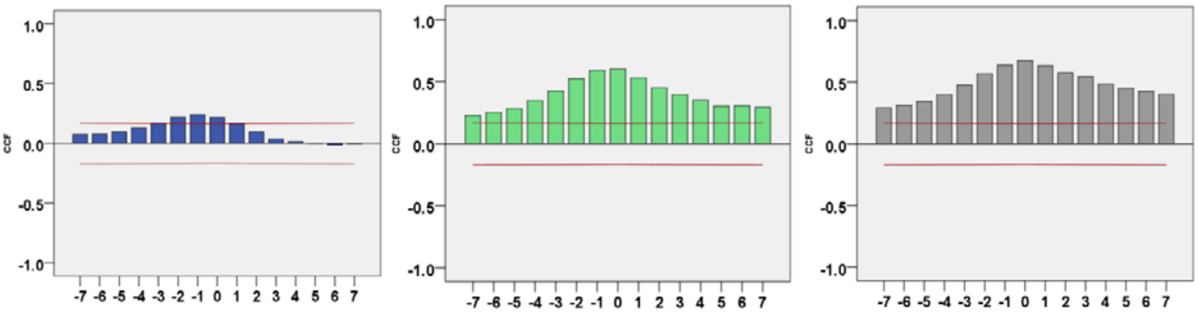

1.0
0.5
0.0
-0.5
-1.0
CCF
-7 -6 -5 -4 -3 -2 -1 0 1 2 3 4 5 6 7


- **Tas**

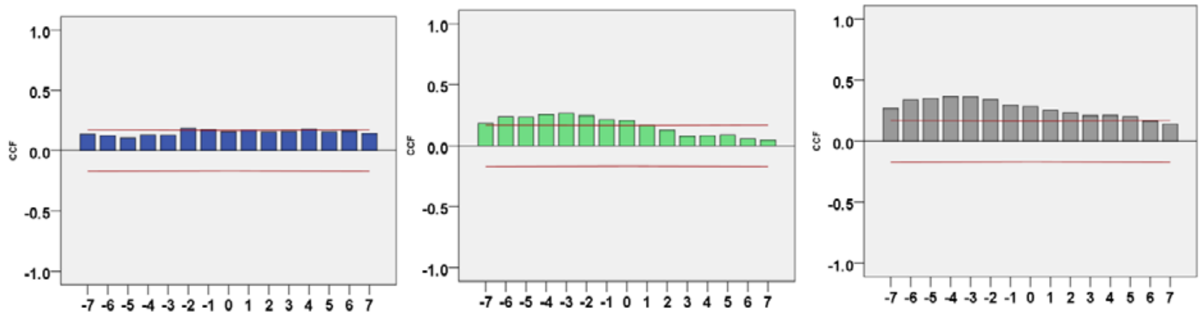

1.0
0.5
0.0
-0.5
-1.0
CCF
-7 -6 -5 -4 -3 -2 -1 0 1 2 3 4 5 6 7


- **Vic**

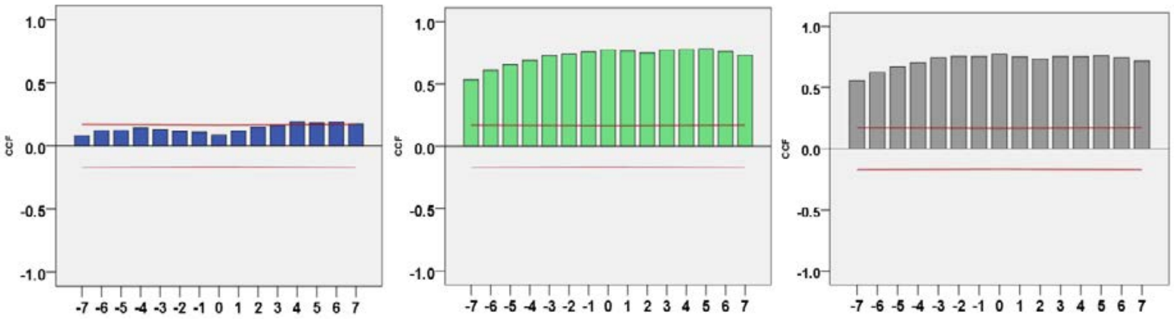

1.0
0.5
0.0
-0.5
-1.0
CCF
-7 -6 -5 -4 -3 -2 -1 0 1 2 3 4 5 6 7


- **WA**

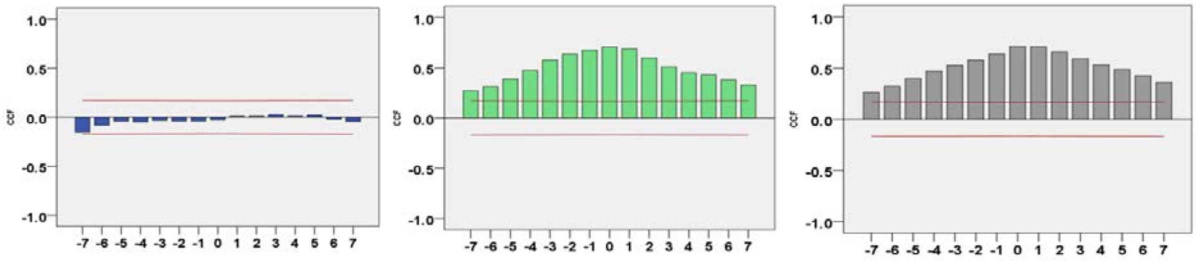

1.0
0.5
0.0
-0.5
-1.0
CCF
-7 -6 -5 -4 -3 -2 -1 0 1 2 3 4 5 6 7

**Figure S3.** Autocorrelation function (ACF) and partial autocorrelation function (PACF) of the residual series of the SARIMA model (2,0,2) (1,0,0).

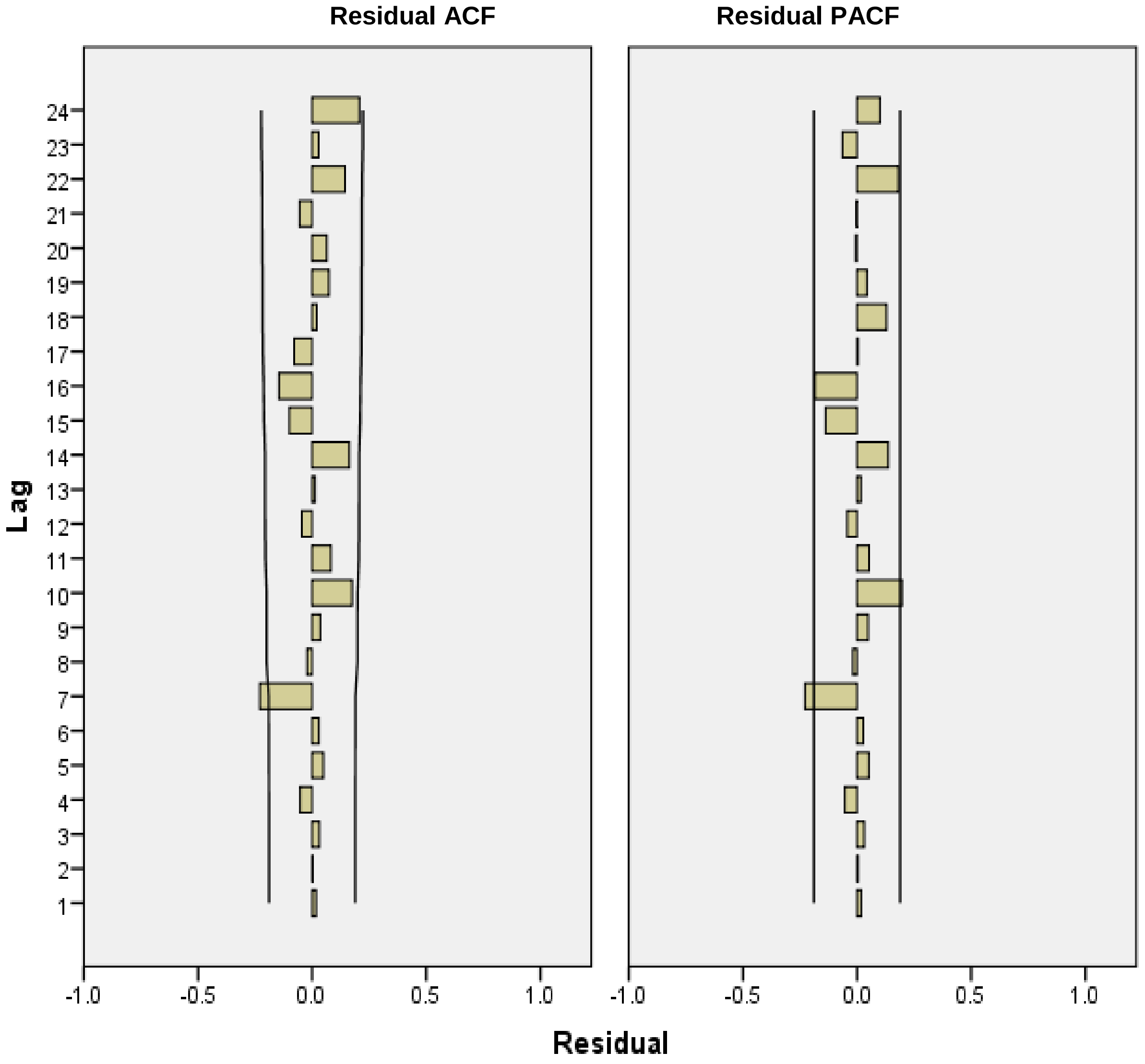

**Table S1.** Spearman's rho values for the monthly GT metrics in Australia from 1 January, 2004 to 31 December, 2015.

| | Google Trends' data | | | | | | | | |
|---|---|---|---|---|---|---|---|---|---|
| **Search term** | AUS | ACT | NSW | NT | QLD | SA | TAS | VIC | WA |
| Pertussis | 0.63** | -0.04 | 0.48** | 0.33** | 0.07 | 0.44** | 0.39** | 0.14 | -0.09 |
| Whooping | 0.81** | 0.54** | 0.71** | 0.61** | 0.55** | 0.60** | 0.23** | 0.81** | 0.74** |
| Whooping cough | 0.82** | 0.64** | 0.71** | 0.51** | 0.52** | 0.63** | 0.18* | 0.81** | 0.77** |

*: p< 0.05; **: p < 0.01.

AUS: Australia; ACT : Australian Capital Territory; NSW: New South Wales; NT: North Territory; QLD: Queensland; SA: South Australia; TAS: Tasmania; VIC: Victoria; WA: Western Australia.

**Table S2.** Time-series cross correlation analysis in 1-2 lag months between the NNDSS pertussis surveillance data and GT from 2004 to 2015.

| Pertussis surveillance data | GT for search term pertussis | | | | | | | | |
|---|---|---|---|---|---|---|---|---|---|
| | Aus | ACT | NSW | NT | Qld | SA | Tas | Vic | WA |
| NNDSS, 2-month preceding | 0.45* | -0.01 | 0.21* | 0.25* | -0.07 | 0.11 | 0.16 | 0.15 | 0.07 |
| NNDSS, 1-month preceding | 0.48* | -0.06 | 0.22* | 0.28* | -0.04 | 0.18* | 0.17 | 0.12 | 0.07 |
| NNDSS, 0-month lagging | 0.55* | -0.06 | 0.29* | 0.32* | -0.00 | 0.23* | 0.16 | 0.10 | -0.03 |
| NNDSS, 1-month lagging | 0.57* | -0.06 | 0.29* | 0.33* | 0.05 | 0.25* | 0.17* | 0.12 | -0.04 |
| NNDSS, 2-month lagging | 0.59* | -0.08 | 0.30* | 0.33* | 0.05 | 0.23* | 0.18* | 0.12 | -0.04 |
| | GT data for search term whooping | | | | | | | | |
| NNDSS, 2-month preceding | 0.63* | 0.46* | 0.58* | 0.42* | 0.51* | 0.45* | 0.13 | 0.74* | 0.60* |
| NNDSS, 1-month preceding | 0.68* | 0.48* | 0.65* | 0.41* | 0.55* | 0.53* | 0.17* | 0.75* | 0.69* |
| NNDSS, 0-month lagging | 0.72* | 0.54* | 0.70* | 0.42* | 0.60* | 0.60* | 0.21* | 0.76* | 0.72* |
| NNDSS, 1-month lagging | 0.70* | 0.47* | 0.66* | 0.41* | 0.59* | 0.59* | 0.21* | 0.75* | 0.68* |
| NNDSS, 2-month lagging | 0.68* | 0.38* | 0.62* | 0.39* | 0.58* | 0.52* | 0.25* | 0.73* | 0.64* |
| | GT data for search term whooping cough | | | | | | | | |
| NNDSS, 2-month preceding | 0.63* | 0.49* | 0.58* | 0.43* | 0.48* | 0.57* | 0.23* | 0.72* | 0.65* |
| NNDSS, 1-month preceding | 0.67* | 0.54* | 0.65* | 0.38* | 0.52* | 0.63* | 0.25* | 0.74* | 0.71* |
| NNDSS, 0-month lagging | 0.72* | 0.61* | 0.69* | 0.36* | 0.57* | 0.67* | 0.28* | 0.76* | 0.71* |
| NNDSS, 1-month lagging | 0.70* | 0.52* | 0.65* | 0.37* | 0.56* | 0.64* | 0.30* | 0.75* | 0.63* |
| NNDSS, 2-month lagging | 0.68* | 0.44* | 0.61* | 0.35* | 0.55* | 0.57* | 0.34* | 0.75* | 0.56* |

*: P<0.05

**Table S3.** Sensitivity and specificity of the SARIMA model (2,0,2) (1,0,0) for pertussis.

| Actual | Predicted | | |
|---|---|---|---|
| | Occurrence | Non occurrence | Total |
| Occurrence | 10 | 3 | 13 |
| Non occurrence | 4 | 19 | 23 |
| Total | 14 | 22 | 36 |

Sensitivity, 10/13 = 77%; Specificity, 19/23 = 83%; and crude agreement, (10+19)/36 = 81%
